# Evaluation of a Validated Food Frequency Questionnaire for Self-Defined Vegans in the United States

**DOI:** 10.3390/nu6072523

**Published:** 2014-07-08

**Authors:** Patricia Dyett, Sujatha Rajaram, Ella H. Haddad, Joan Sabate

**Affiliations:** 1 Department of Agricultural Economics and Extension, University of the West Indies, St. Augustine, Trinidad and Tobago; E-Mail: patricia.dyett@sta.uwi.edu; 2 School of Public Health, Loma Linda University, Loma Linda, CA 92350, USA; E-Mails: ehaddad@llu.edu (E.H.H.); jsabate@llu.edu (J.S.)

**Keywords:** vegans, food frequency questionnaire, diet recalls, nutrient intake, Dietary Reference Intakes, vegan questionnaire, vegetarians, vitamin D, vitamin B_12_

## Abstract

This study aimed to develop and validate a *de novo* food frequency questionnaire for self-defined vegans in the United States. Diet histories from pilot samples of vegans and a modified ‘Block Method’ using seven selected nutrients of concern in vegan diet patterns, were employed to generate the questionnaire food list. Food frequency responses of 100 vegans from 19 different U.S. states were obtained via completed mailed questionnaires and compared to multiple telephone-conducted diet recall interviews. Computerized diet analyses were performed. Correlation coefficients, *t*-tests, rank, cross-tabulations, and probability tests were used to validate and compare intake estimates and dietary reference intake (DRI) assessment trends between the two methods. A 369-item vegan-specific questionnaire was developed with 252 listed food frequency items. Calorie-adjusted correlation coefficients ranged from *r =* 0.374 to 0.600 (*p <* 0.001) for all analyzed nutrients except calcium. Estimates, ranks, trends and higher-level participant percentile placements for Vitamin B_12_ were similar with both methods. Questionnaire intakes were higher than recalls for most other nutrients. Both methods demonstrated similar trends in DRI adequacy assessment (e.g., significantly inadequate vitamin D intake among vegans). This vegan-specific questionnaire can be a useful assessment tool for health screening initiatives in U.S. vegan communities.

## 1. Introduction

Interest in the vegetarian lifestyle has increased significantly in parts of North America over recent decades [1,2], attributed largely to the beneficial health effects of plant-based diets [3,4,5,6]. With this increasing trend, careful consideration should be given to efficient assessment and surveillance of vegetarian diet patterns, particularly with respect to specific nutrient profiles. Over the years, several important benefits of vegetarian diets has been identified, as well as specific components of plant-based diets that may account for these benefits [7,8]. It is, therefore, no surprise that the position of the American Dietetic Association suggests that such diets, when appropriately planned, are healthful, nutritionally adequate, and provide health benefits in the prevention and treatment of certain diseases [9].

Despite the health and nutritional benefits however, there remains concern of specific nutrient inadequacy, particularly for vegans who practice an exclusively plant food diet void of all animal products. Some nutrients seem to be of more concern than others: protein, *n*-3 fatty acids, calcium, zinc, iron, vitamin D and vitamin B_12_ [7,8]. These important nutrient concerns justify the need for appropriate nutrition assessment methods specific to the vegan population. 

Apart from direct clinical or biochemical measures, which are lacking for many nutrients, the most common tools of nutrition assessment are indirect and include the food record, diet histories, 24-h diet recalls, and food frequency questionnaires (FFQs). Among these, the FFQ emerges as an effective, easily administered, inexpensive tool [10,11]. Many FFQs have been developed or modified to assess adequacy of nutrient and diet patterns, and to determine association between diet and chronic disease risk [12,13,14,15,16]. To the best of our knowledge, no study has developed or validated a FFQ specific to vegan diet assessment in the United States.

We therefore proposed that a well-developed FFQ specific to the vegan population could produce performance outcomes comparable to other methods of nutrition assessment and thus can be effectively used by health and nutrition professionals in addressing the nutrient-related concerns of the plant based diet patterns among the region’s vegan clientele.

## 2. Experimental Section

The study had three phases: (i) development of a quantitative FFQ with appropriate food list, portion size options, and frequency options, obtained from a pilot sample of U.S. vegans and using a modified version of the Block Method [10], along with support questions; (ii) relative validation of the FFQ by comparing intakes of protein, *n*-3 fatty acids, iron, calcium, zinc, vitamin B_12_ and vitamin D, with repeated 24-h diet recalls; and (iii) comparison of the dietary assessment data with relevant Dietary Reference Intake (DRI) values to compare trends between methods. Approval for the study proposal was obtained from the Institutional Review Board of Loma Linda University, Loma Linda, CA, USA, where the study was conducted. 

### 2.1. Questionnaire Development

#### 2.1.1. Pilot Sample of Vegans

Recruitment was done via the Vegetarian Resource Group, a non-profit organization based in Maryland, USA, which is comprised of members practicing a vegetarian lifestyle and committed to educating the public on vegetarianism. Recruitment resulted in a convenience sample of 50 self-defined vegans who volunteered to provide diet histories. Based on these, the major contributing food sources in their diets for proteins, *n*-3 fatty acids, vitamin B_12_, vitamin D, calcium, iron and zinc were ascertained. Though self-defined, the pilot sample met our minimum study criteria of consuming no meat, fish, or poultry and using dairy products or eggs less than once per month.

#### 2.1.2. Food List

From the collective food data obtained from the pilot sample of vegans, 330 single foods were reported as being used on a regular basis (>twice/month). The 330 foods were entered into the Nutrition Data System for Research (NDS-R) Database version 4.06_34, Regents of the University of Minnesota (Minneapolis, Minnesota, USA). A modified version of the Block Method [10,17] was used to generate a list of foods that contributed 80% of each nutrient of interest. Of the initial 330 single foods, 180 single foods were identified as contributing intake of 80% of each of the nutrients of interest. These foods formed the basis for the vegan food list. A focus group of dietitians and public health professionals were then consulted concerning the list generated. Modifications and additions to the food list were made accordingly and generated the following food list categories: (1) peas and beans; (2) nuts, seeds, nut butters; (3) vegetarian meat analogs, cheese and egg substitutes, mixed foods such as vegan lasagna; (4) fats, oils and salad dressings; (5) breads; (6) cooked cereals and grains; (7) ready-to-eat cereals; (8) leafy vegetables; (9) non-leafy vegetables; (10) fruits and fruit juices; (11) milk substitutes; other beverages such as herbal teas, coffee, and alcohol; (12) salty snacks such as potato chips and popcorn; (13) desserts, sweets, and sweeteners. A total of 252 listed close-ended food item questions were generated among the categories. For example, under the cooked cereal category, specific cereal types were listed such as oatmeal, barley, spelt, *etc.* Food frequency sub-questions of an open-ended nature were also included (e.g., three spaces for other cooked cereals used). Support questions on supplement use, demographics, anthropometrics, health status, meal practices, and lifestyle, were also included. The modified list was then entered into the NDS-R version 5.0_35, to benefit from additional listings of vegetarian meat analogs found in the updated diet analysis program.

#### 2.1.3. Questionnaire Format

The general format of the questionnaire was then addressed to include instructions, portion size options, frequency options, and support questions. Apart from typical questionnaire instructions, the food frequency section of the questionnaire was introduced with the instruction that all intake responses should reflect consumption patterns over the past nine months. This was in an effort to capture vegan intake patterns from both long-standing and recently practicing vegans. 

Food frequency question portions were selected and coded based on the three most frequently reported portion sizes for each food item among the pilot sample. For example, the most frequently reported portion sizes for nuts and seeds were 1/8 cup, 1/4 cup, and 1/2 cup. Each of these unit measures was given a weight factor with the middle portion as the reference weight. Therefore, for nuts, the middle portion unit, (1/4 cup) was coded and given a weight of [2 = 1], 1/8 cup was given a code and weight of [1 = 0.5] and 1/2 cup was given a code and weight of [3 = 2]. Six frequency options were also derived from most frequently reported pilot survey frequencies. This falls within typical frequency ranges of other food frequency questionnaires [18]. These were coded as follows: [4 = 0] for never or rarely; [5 = 0.75] for two to four times per month; [6 = 2.5] for two to three times per week; [7 = 5] for four to six times per week; [8 = 7] for once per day; and [9 = 14] for two to three or more times per day. While the latter option indicated a wide range of two to three or more times per day, it was coded for twice per day since two times per day was the most frequently reported frequency in the pilot survey.

To enhance interpretation from food frequency estimates, and to capture possible screening tool properties, support questions on vitamin, mineral, and herbal supplement intake, demographics, anthropometrics, self-reported diagnosed health status, lifestyle, and meal practices, were also included in the questionnaire. Some of these included both open-ended and closed-ended questionnaire format. The questionnaire was then pilot-tested on a sub-sample (*n =* 10) of the fifty vegans. Final modifications were then made to the content and format of the questionnaire. 

### 2.2. Validation of the FFQ

#### 2.2.1. Validation Study Sample

To arrive at the sample size, we first established correlation criteria. Because previous studies demonstrate that correlation coefficients for validation studies usually fall within the range of 0.3 to 0.7 [18,19,20], we assessed correlation between the FFQ and the diet recalls using a hypothesis of H_O_: *r <* 0.3 and H_A_: *r* ≥ 0.3. Therefore the minimum sample size was calculated to be *n =* 91 for a desired lower limit of one-sided 95% confidence interval at *r =* 0.3 and a population correlation of 0.45. 

The target population was generated from telephone, email, and post mail address contact lists provided by the Vegetarian Resource Group, other vegetarian organizations, churches, and recruitment fliers posted at the host university. All persons on the generated list were mailed consent forms and questionnaires. Based on returned consent forms and questionnaire responses, participants were included in the analysis if they were within the age range of 25 to 75 years and met the study’s vegan criteria. Vegans were defined as individuals who consumed no meat, fish, or poultry, and who consumed dairy products or eggs less than once per month. A convenience sample of vegans was selected from across 19 U.S. States.

#### 2.2.2. Nutrient Intake Data from the FFQ and Diet Recalls

The questionnaire administration at baseline was followed by three non-consecutive, unannounced telephone diet recalls over a nine-month period. Questionnaires were completed and returned within a three week period. Analyzed dietary intake data from the food frequency responses were obtained from the Nutrition Data Systems for Research software version 5.0_35. Food frequency questionnaire intake data were entered along with diet recall data into a statistical analysis program and calculated as follows:

FFQ nutrient intake score per day = (coded PF × coded FF × WF per question)/7
(1)

where “PF” represents portion factor, “FF” represents frequency factor, and “WF” represents weight factor per food frequency question which is the unit amount of the nutrient for the middle portion size option. 

Two weekday recalls and one weekend recall were obtained from each subject in the spring, summer, and fall to capture seasonal foods. Two trained diet interviewers collected the recall data via telephone and prompted for specific information such as number and size of utensil, cup, spoon, *etc.* that the respondents used per food item reported. Nutrient analyses for the diet recalls were then performed with the NDS-R version 5.0_35. To obtain an appropriate ratio of weekend days to weekdays as referred to by other researchers [21], a weighted mean intake was computed from the three diet recalls in order to represent intake over a week. The weighted average intake per day was then calculated as follows:

Weighted diet recall average for each nutrient per day = (WE × 2 + WD × 5/2 + WD × 5/2)/7
(2)

where “WE” represents weekend intake of nutrients and “WD” represents weekday intakes.

#### 2.2.3. Statistical Validation

The nutrient intakes from the two assessment methods were log-transformed to improve normality using natural log_e_(ln(*x* + 1)) and adjusted for energy using the residual regression method with energy intake as the independent variable and nutrient intakes as the dependent variables. Pearson’s correlation coefficients and standardized correlations (crude intake) were used for analyses between methods. Signed-ranks tests, quartile ranking, and cross-tabulation of participant intake percentile placement agreements were used to analyze the untransformed intake estimates in order to evaluate whether FFQ and diet recall intakes were similarly ranked per each nutrient and participant. Paired samples *t*-tests were also used to compare transformed intake estimates between methods. The Predictive Analytics SoftWare (PASW) Statistical Package, version 18.0 software (SPSS Incorporated, Chicago, IL, USA) was used for all analyses.

### 2.3. Dietary Reference Intake Comparison

One-sample *t*-tests were used to compare mean nutrient intake from the two methods against the mean of the most recent Dietary Reference Intake (DRI) values based on age and sex of the study participants [22,23]. For protein, vitamin B_12_, iron, zinc, calcium, and vitamin D, the DRI used was the mean of the Recommended Dietary Allowances (RDA) value for the study group. For *n*-3 fatty acids, the mean of the Adequate Intake (AI) value was used. Prevalence of adequacy was also estimated for relevant nutrients using the probability approach, also referred to as the Estimated Average Requirement (EAR) cut-point method [23]. EAR is the average daily nutrient intake level estimated to meet the requirements of half the healthy people in a group; and the EAR cut-point method has been used to identify the percentage of subjects with intakes below or above the EAR value (used as the cut-point) for the nutrient [24]. 

## 3. Results

### 3.1. The Vegan Questionnaire

A final 369-item questionnaire was produced, containing 13 food list categories, 252 close-ended food item questions, 49 open-ended food list sub-questions among the categories, three portion size options, six frequency options, and 68 support questions. The food frequency questionnaire for vegans is included in the Supplementary Information. 

### 3.2. Questionnaire Validation

From an original convenience sample of 132 recruited vegans, 15 questionnaires were not returned; eight participants discontinued the study for personal reasons; two did not meet the vegan criteria, and seven were lost to follow-up after the first diet recall. Therefore, 100 subjects (24 males and 76 females) satisfactorily completed both the questionnaire and the 24-h recalls. Table 1 describes the characteristics of the validation phase participants. 

**Table 1:** Demographic data for vegan study participants.

Vegan Years ^a, b^	<1 (*n =* 5)	1–3 (*n =* 19)	4–10 (*n =* 42)	>10 (*n =* 33)	Blank ^c^ (*n*)	Totals (*n*)
*Race*						
Caucasian	5	11	30	25	-	71
Black	0	4	4	4	1	13
Asian	0	3	5	2	-	10
Other	0	1	3	2	-	6
*BMI* ^b^						
<18.5	0	0	5	2	-	7
18.5–24.9	4	10	32	25	-	71
25–29.9	1	7	4	5	-	17
≥30	0	2	1	1	-	4
*Age*						
25–39	2	12	21	9	-	44
40–59	2	5	14	16	-	37
60–75	1	2	7	8	1	19
*Education* ^b^						
High school	1	4	0	1	-	6
Some college	2	3	6	7	-	18
Bachelor’s	0	4	12	12	-	28
Graduate level	2	8	24	13	-	47

^a^ The length of time the participant has been a vegan; ^b^ Missing data for one participant; ^c^ Participant left the *vegan years* item blank on the survey.

Table 2 presents energy-adjusted and non-adjusted correlation coefficients comparing log-transformed and standardized nutrient intake values from the questionnaire for vegans and three 24-h diet recalls. Pearson’s correlation coefficients improved for all the nutrients when adjusted for energy, and were relatively high for six of the nutrients of interest (*r* = 0.374 to *r* = 0.600). With the standardized correlation analysis, four of the seven nutrients of interest were significantly correlated at *r* ≥ 0.305. Calcium intake estimates between the two assessment methods were not significantly correlated.

**Table 2:** Correlation coefficients for nutrient intakes from the vegan food frequency questionnaires (FFQ) and three 24-h diet recalls.

Nutrients	Non-Adjusted Correlations ^ab^	*p*-Values	Kcal-Adjusted Correlations ^ac^	*p*-Values	Standardized Correlations ^d^	*p*-Values
Protein (g)	0.365	<0.001	0.374	<0.001	0.291	0.003
*n*-3 FA (g)	0.501	<0.001	0.600	<0.001	0.468	<0.001
Calcium (mg)	0.128	0.206	0.191	0.057	−0.031	0.758
Iron (mg)	0.371	<0.001	0.449	<0.001	0.296	0.003
Zinc (mg)	0.399	<0.001	0.506	<0.001	0.332	0.001
Vitamin D (µg)	0.391	0.001	0.464	<0.001	0.305	0.002
Vitamin B_12_(µg)	0.335	0.001	0.403	<0.001	0.339	0.001

*n =* 100; ^a^ Nutrient intakes have been log-transformed using log_e_ to improve normality; ^b^ Pearson *r* correlations for nutrient values unadjusted for energy; ^c^ Pearson *r* correlations for energy-adjusted nutrients using the residual regression method; ^d^ Standardized (*z* score) correlation coefficients of crude nutrient intakes between methods.

Table 3 shows untransformed intake results from the signed-ranks test and quartile ranking. From the signed ranks test it was observed that for six of the nutrients, FFQ intake significantly exceeded DR intake for more than 60 participants; and for about 50 vegans there was no significant difference in vitamin B_12_ intake. Quartile rankings showed that each intake percentile cut point was higher for the FFQ than the DR for all nutrients except for the 50th percentile of vitamin B_12_ intake.

**Table 3:** Comparison of nutrient intakes between the vegan FFQ and three 24-h diet recalls using signed ranks test and quartile ranking for evaluation.

Signed Ranks Test for DR-FFQ ^a^				*p*-Value	FFQ Intake Quartiles ^c^			DR Intake Quartiles ^c^		
Nutrient	Ranking ^b^	*N*	Mean Rank		25th	50th	75th	25th	50th	75th
Protein (g)	Negative	76	56.49	<0.001	57.31	80.51	124.24	45.19	57.13	69.46
	Positive	24	31.54	-	-	-	-	-	-	-
	Ties	0	-	-	-	-	-	-	-	-
*n*-3 FA (g)	Negative	76	53.89	<0.001	1.81	3.29	4.69	1.17	2.16	3.27
	Positive	24	39.75	-	-	-	-	-	-	-
	Ties	0	-	-	-	-	-	-	-	-
Calcium (mg)	Negative	84	54.23	<0.001	755.46	1051.64	1764.32	477.77	623.77	861.80
	Positive	16	30.94	-	-	-	-	-	-	-
	Ties	0	-	-	-	-	-	-	-	-
Iron (mg)	Negative	75	54.72	<0.001	17.12	21.20	32.31	13.58	17.34	21.57
	Positive	25	37.84	-	-	-	-	-	-	-
	Ties	0	-	-	-	-	-	-	-	-
Zinc (mg)	Negative	76	56.82	<0.001	9.02	11.72	17.05	6.51	8.32	11.68
	Positive	24	30.50	-	-	-	-	-	-	-
	Ties	0	-	-	-	-	-	-	-	-
Vitamin D (µg)	Negative	61	52.24	0.013	0.77	1.99	3.30	0.43	1.48	2.69
	Positive	38	46.41	-	-	-	-	-	-	-
	Ties	1	-	-	-	-	-	-	-	-
Vitamin B_12_(µg)	Negative	51	49.73	0.970	1.22	2.15	5.13	1.14	2.72	4.44
	Positive	49	51.31	-	-	-	-	-	-	-
	Ties	0	-	-	-	-	-	-	-	-

*n =* 100; degrees of freedom = 99; ^a^ Rank for crude diet recall nutrient intake minus crude food frequency questionnaire nutrient intake; ^b^ Ranking: *Negative Ranks* mean diet recall nutrient intake < questionnaire nutrient intake; Positive Ranks mean diet recall nutrient intake > questionnaire nutrient intake; Ties mean diet recall nutrient intake = questionnaire nutrient intake; ^c^ Crude nutrient intake values from FFQ and diet recalls used for quartile cut point assessment.

**Table 4:** Nutrient intake comparisons for the vegan FFQ and diet recalls using cross-tabulated participant percentile placement summaries and paired samples *t*-tests for evaluation.

Nutrients	Participant Interquartile Intake Placement Agreements for FFQ *vs.* DR ^a^				Paired Differences ^b^				
	<25th	25th–50th	50th–75th	>75th	Mean ^c^	SD	*p*-Value	95% CI Limits	
Protein (g)	12	7	8	8	0.36	0.55	<0.001	0.25	0.47
*n*-3 FA (g)	10	9	9	12	0.32	0.53	<0.001	0.21	0.42
Calcium (mg)	10	6	7	5	0.63	0.78	<0.001	0.47	0.78
Iron (mg)	13	10	6	10	0.28	0.48	<0.001	0.18	0.37
Zinc (mg)	10	7	3	13	0.34	0.47	<0.001	0.24	0.43
Vitamin D (µg)	13	8	11	12	0.16	63	0.012	0.04	0.29
Vitamin B_12_ (µg)	7	4	8	12	−0.02	0.77	0.835	−0.17	0.14

*n =*100; degrees of freedom = 99; ^a^ Number of similarly placed vegan participants between the FFQ and diet recalls based on interquartile cross-tabulations summarized from the diagonal plane; ^b^ FFQ intake minus Diet Recall intake for the selected nutrients; ^c^ Mean difference between log_e_-transformed intakes for FFQ minus diet recall.

In Table 4 the paired samples *t*-test also demonstrated no significant difference in estimated intake for vitamin B_12_ between the FFQ and the diet recalls (*p* = 0.835); and for all other nutrients the FFQ intakes were significantly higher than those of the diet recalls. For each of the nutrients, the cross-tabulations for similarly placed vegan participants within intake percentiles on the diagonal plane showed similarities for less than 45 of 100 participants between methods. Calcium and vitamin B_12_ cross-tabulations had the least similarities while *n*-3 fatty acids and vitamin D had the most (Table 4). However, from the quartile totals for the FFQ and DR (not shown), it was observed that for calcium, protein, iron, and zinc, participants were evenly distributed (*n* = 25) among each quartile for both methods; *n*-3 fatty acids had agreement in totals for the 4th quartile; vitamin D demonstrated agreement in totals for the 1st and 2nd quartiles; and for vitamin B12, quartile totals agreement was observed for the 3rd and 4th quartiles.

### 3.3. Dietary Reference Intake Trends

Although actual intake estimates were significantly different between the two assessment methods for most of the nutrients, both assessment methods showed similar intake trends when compared to the Dietary Reference Intake recommendations. In Table 5, one sample *t*-tests showed that mean vitamin D intake among the vegans was significantly lower than the mean recommended intake for both assessment methods. However, both methods demonstrated a trend of intakes higher than the DRI values for most of the other nutrients. Two exceptions were noted with the diet recalls: where calcium was observed to be significantly lower than the DRI value and zinc intake was not significantly different from the DRI recommendation. The probability approach showed that 100% of the study sample demonstrated inadequate intakes of vitamin D via both assessment methods; and 74% demonstrated inadequate intakes of calcium via the diet recalls (Table 5).

**Table 5:** Comparison of mean nutrient intake trends from the vegan FFQ and three 24-h diet recalls with Dietary Reference Intake values using one sample *t*-test and probability approach for evaluation.

Nutrients	FFQ *vs.* DRI One Sample *t*-Test Values					*p*-Value	Probability Approach	
	FFQ Intake ^a^	SE	DRI ^b^	Mean ^c^	SE		EAR ^d^	% Adequacy ^e^
Protein (g)	94.74	5.35	48.40	46.34	5.37	<0.001	42.70	89
*n*-3 FA (g) ^f^	4.01	0.29	1.22	2.79	0.29	<0.001	--	--
Calcium (mg)	1520.28	152.81	1048.00	472.28	153.97	0.003	848.00	65
Iron (mg)	25.94	1.42	13.30	12.64	1.51	<0.001	6.88	99
Zinc (mg)	13.92	0.73	8.72	5.20	0.76	<0.001	7.42	86
Vitamin D (µg)	2.28	0.18	15.15	−12.87	0.20	<0.001	10.00	0
Vitamin B_12_ (µg)	3.31	0.31	2.40	0.91	0.31	0.004	2.00	51
*Diet Recalls vs. DRI One Sample t-Test Values*								
**Nutrients**	**DR Intake ^g^**	**SE**	**DRI ^b^**	**Mean ^h^**	**SE**	***p*-value**	**EAR ^d^**	**% Adequacy ^e^**
Protein (g)	60.44	2.28	48.40	12.04	2.24	<0.001	42.70	77
*n*-3 FA (g)	2.65	0.25	1.22	1.43	0.25	<0.001	--	--
Calcium (mg)	681.93	30.41	1048.00	−366.07	32.63	<0.001	848.00	26
Iron (mg)	18.39	0.73	13.30	5.08	0.89	<0.001	6.88	98
Zinc (mg)	9.27	0.40	8.72	0.55	0.39	0.166	7.42	62
Vitamin D (µg)	1.82	0.16	15.15	−13.33	0.19	<0.001	10.00	0
Vitamin B_12_ (µg)	3.24	0.27	2.40	0.84	0.27	0.003	2.00	62

*n =*100; degrees of freedom = 99; ^a^ FFQ intake represents the antilog of the log-transformed nutrient intake scores from the FFQ; ^b^ DRI represents the antilog of the log-transformed Dietary Reference Intake value for each nutrient based on age and gender for all subjects; using either Recommended Dietary Allowances (RDA) or Adequate Intakes (AI) where applicable: Protein (RDA); *n*-3 FA (AI); Calcium (RDA); Iron (RDA); Zinc (RDA); vitamin D (RDA); vitamin B (RDA); ^c^ Mean difference between FFQ intakes and DRI values; ^d^ Mean Estimated Average Requirement (EAR) cut point values based on age and gender of participants; ^e^ Percentage of vegans with crude nutrient intakes above the EAR values for the probability approach; ^f^ No Estimated Average Requirement reference value available; ^g^ DR intake represents the antilog of the log-transformed nutrient intakes from 3 weighted diet recalls; ^h^ Mean difference between diet recall intakes and DRI values.

## 4. Discussion

Our study accomplished the *de novo* development of the first food frequency questionnaire specific to vegan diet patterns in the United States. An overall evaluation of the questionnaire highlights the successful genesis of this target-tailored nutrition health screening tool with an initial food list of items that were regularly consumed by the piloted vegans and which contributed 80% of selected nutrients of particular concern in the vegan diet since no animal products are used: protein, *n*-3 fatty acids, vitamin B_12_, vitamin D, calcium, iron, and zinc. Nutrient intake from the FFQ demonstrated moderate to high relative validity for most of the nutrients of interest when correlated with intakes from repeated 24-h diet recalls, though actual intake estimates from the FFQ were higher. When intake estimations were compared to DRI recommendations, the trend observed by both assessment methods was that the majority of vegans were found to have inadequate intakes of vitamin D, but adequate intakes for most other nutrients. Specific evaluation of the choice, development, validation, and performance of the vegan questionnaire, are discussed in the following sections.

### 4.1. Evaluation of the Choice of Intake Assessment Tool

Assessment of nutrient intake can include the use of biochemical measures as well as computation of the energy and nutrient content of foods using values obtained from food composition tables, computer based diet analysis programs, or even direct chemical food analysis in a laboratory [25]. Common diet assessment methods that facilitate nutrient intake estimations are diet records, FFQ, diet histories, and 24-h recalls. Because we desired a method or instrument that would be particularly unique to vegans, the FFQ emerged as the only assessment instrument that can be crafted or manipulated in such a way as to capture reported intakes relevant to the target population.

### 4.2. Evaluation of the De Novo Development of the Vegan FFQ

Various studies have used FFQs along with other assessment methods for evaluating different aspects of the vegan lifestyle. The majority of these have been primarily generic or modified questionnaires used not just for vegans but for other vegetarian and omnivorous diet groups as well [26,27,28,29]. The importance of designing questionnaires specific to a target population has been shown [30,31,32]. This approach is particularly important when targeting the vegan population because of their unique diet patterns and their use of certain foods other than fruits and vegetables, which are not habitually consumed by other diet groups [33]. Some of these foods include meat analogs, cheese, and egg substitutes, and alternative milk products like soy, rice and almond milks, non-dairy salad dressings, and particular cereal brands [34].

#### 4.2.1. Food List Development

To compile the food list for the questionnaire, a modification of the method developed by Block *et al.* [10] was used. Our study looked for an 80% nutrient contribution instead of 90% contribution, since we were dealing with a more limited list of nutrient contributors due to the absence of animal products. According to Willett [35], a further modification includes tallying foods reported via open-ended methods in a sample of the study population. This serves to capture information regarding familiar names or descriptions of foods, particularly for ethnic or unique diet groups, and a tally of portion size can simultaneously be made. For our study, a mean portion size was obtained for each food item used in the vegan sample from a tally of the reported portion sizes. Since some items in a collapsed group might be more frequently consumed than others in the same grouping, we chose not to have too many food groupings, as this can be a challenge for respondents to estimate [35,36]. In fact after pilot testing, and focus group consultation, the original food list in our study was expanded by using more individual line items under most of the food groupings. As such we anticipated a higher degree of intake overestimation than underestimation due to the number of single line items and sub-divisions in the FFQ as opposed to groupings [18]. This may account for the higher intake estimates observed in the FFQ when compared to the 24-h recalls. However, some research has shown that validity of a FFQ is increasingly comparative to diet recalls as the number of food and beverage items on the FFQ food list increases [37,38].

#### 4.2.2. Questionnaire Format

For the frequency and portion size sections of the questionnaire, quantitative options were used since estimated intake calculations were being made. The semi-quantitative format was not used because we felt that respondents who used portion sizes other than the pre-determined ones, would have had additional mental calculations to perform and, thereby, lower the degree of accuracy in estimations. It has been shown that using standard or pre-determined portions rather than reported portion size data can serve to simplify questionnaires and decrease cost of data collection [39]. The vegan questionnaire was structured for ease of coding. As such, a close-ended frequency format instead of a participant-reported frequency format was used. It cannot be fully determined whether this may have impacted the study results as some researchers are of the opinion that using participant-reported frequencies may yield higher coefficients and better quartile agreements than close-ended, pre-coded frequency categories [40]. Our study demonstrated fairly high correlation coefficients and high agreement among quartile totals. With respect to the frequency options, it was surmised that since individuals typically do not use an item more than three to four times per day, we used a range of six options, with “two to three+ times/day” as the highest frequency option, and “Never or Rarely” as the lowest frequency. Questions on demographics, lifestyle, health, and meal patterns, were also included in the questionnaire to add qualitative support for quantitative evaluation and validation outcomes.

### 4.3. Evaluation of the FFQ Validation with Diet Recalls

The reference method used in the study was repeated 24-h diet recalls. This was used in an attempt to capture habitual intake without the recording bias and burden as is often observed with the use of diet records [35]. Although diet recalls may tend to share common errors with FFQs in terms of memory and the same food composition table used for both, other studies have reported acceptable use of the diet recall as an instrument of reference comparison [41,42,43,44]. The nutrient intake values for both the FFQ and the diet recalls in our study were adjusted for energy since this can help to reduce a reasonable degree of some measurement error [45,46]. Standardized correlation analysis was also used in an effort to reduce the correlation attenuation due to between-person variation. However, measurement error from over-reporting, as is typically seen with questionnaires [45], and under-reporting, as commonly occurs with diet recalls [21], may partially account for the low correlation coefficient values observed for calcium using both the Pearson’s correlation (*r* = 0.191) and the standardized correlation analysis (*r* = −0.031). It was noted that only a few categories of fortified foods were reported as the main source of calcium from the diet recalls, for this group of vegans. As such, when some of these participants did not consume such foods on a daily basis, especially when one or more of those limiting days happened to be a diet recall day, disparity between the diet recalls and the FFQ was very probable since the FFQ naturally allowed for a more comprehensive report of the typical calcium-containing foods consumed by the target population. Correlation coefficients are commonly used in validation or comparison studies; and the moderate to high values observed for the other analyzed nutrients in our study are comparable with values typically reported for similar studies [18,19,20,43]. 

In our study, the paired samples *t*-tests and the signed rank tests resulted in significantly higher FFQ nutrient intake values than the diet recall intake values for all the analyzed nutrients, except vitamin B_12_. These observed differences may also be explained by over-reporting/under-reporting between assessment methods. However, the observed intake similarity between methods for vitamin B_12_ may have resulted from a more conscious emphasis by this group of vegans to ensure daily and consistent intake of this nutrient, since among all the nutrients of concern to vegans, vitamin B_12_ is commonly known to be the one least available naturally from plant sources. Participant placement agreement between methods was observed for the highest (3rd and 4th) vitamin B_12_ intake quartile totals.

### 4.4. Evaluation of FFQ-Generated DRI Assessment Trends

The vegan diet completely omits food products from the array of animal sources like meat, poultry, fish, eggs, and dairy that are rich in some of the selected nutrients. Hence the reason for concern that inadequate intakes of nutrients such as protein, calcium, vitamin D, vitamin B_12_, *n*-3 fatty acids, iron, and zinc may exist within the vegan population since plant foods may not provide adequate amounts of these nutrients [9,47], or the nutrients may not be as bioavailable from plants due to the inhibiting presence of inhibitors like phytates, oxalates, and fiber [9,47]. Therefore comparative assessment of nutrient intake with DRI standards is a necessary health screening endeavor in vegan populations. In our study, both the FFQ and the diet recalls demonstrated parallel trends in discriminating intake adequacy when compared to DRI values. Both methods demonstrated 0% adequacy with respect to vitamin D intake; and both methods demonstrated intakes above standard recommendations for all other analyzed nutrients, except calcium. Other studies have observed inadequate intake and nutrient status trends in vegans for calcium and vitamin D [48,49,50]. However, unlike our findings, some studies have not been able to observe acceptable proportions of adequacy for vitamin B_12_ [51], zinc [52], and *n*-3 fatty acids [53,54].

### 4.5. Study Strengths and Limitations

The authors of this study acknowledge limitations associated with conducting a study of this nature. Participants were selected from a convenience sample, which may not entirely represent the target population. However, the vegan study sample was obtained from across several different states and from a diverse background of ethnicities, age groups, genders, health status, meal behaviors, motivation factors, and lifestyle practices. The participants’ demographic characteristics were also comparable to the pilot sample particularly with respect to age, race and gender ratios; but a greater proportion of the pilot sample practiced veganism for more than 10 years. The final sample size used in this study was conservative (*n* = 100). Vegans practice unique dietary and lifestyle habits and therefore represent a relatively small percent (1.6%) of the U.S. population [1]. The minimum sample size calculated for our study was 91. There is agreement for the use of sample sizes for validation studies with 100 or less subjects [18,31,35]. In addition to the size of the sample, the study timeline, the different time zones, and the available resource allowances facilitated data collection of a maximum of three diet recalls in our study. Some researchers suggest this is the minimum number of diet recalls needed to represent usual intake for some nutrients [35,42]. There is support of the use of three diet replicates per subject in other studies [18,37,41,42,44,55]. However, as was noted by Streppel and colleagues [44], we also realize that a larger number of recall replicates helps to reduce the daily variation of infrequently consumed foods. Additionally, only one entry was made per questionnaire and no reproducibility measures were taken due to study timeline constraints. 

Regarding nutrient intake comparison with the DRIs, we realize that use of the EAR cut-point probability approach is designed only for nutrients with established EAR values. Therefore six of the seven nutrients of interest were able to be analyzed by this method. Our analyses focused on nutrients obtained from food intake and did not take into primary account the contribution of various supplements that may have been taken by some of the vegans. Nonetheless, relevant support questions on supplement use added qualitative insight. For example, of the vegans who reported on supplement use, 80 reported never or rarely using vitamin D supplements; while 25 and 38 reported using calcium or multivitamin supplements respectively, on a regular basis (≥2 per week). The use of indirect methods of nutrient assessment naturally limited our assessment scope, but use of more direct assessment methods for nutrient intake and status, such as biomarkers for seven nutrients for 100 subjects from across several different states, was not a feasible option within our study context.

## 5. Conclusions

Evaluation of the originally developed questionnaire for U.S. vegans demonstrated moderate to high validity for the majority of analyzed nutrients when compared to multiple 24-h diet recalls. When compared to the DRIs, both methods found vitamin D intake trends to be below individual requirements for all the vegan participants. Except for calcium, intake estimates from both methods were found to be higher than DRI values for all other analyzed nutrients. We conclude that this FFQ can have useful application for vegans residing in the United States. Our study results also prompt recommendation that all persons practicing a vegan lifestyle should ensure diets containing adequate natural, fortified, or supplemental sources of all the vitamins, minerals, and macronutrients of interest; particularly, vitamin D and calcium. This study demonstrates that carefully developed questionnaires have great potential for nutrition assessment and health screening within target populations.

## Supplementary Files

Supplementary File 1 Supplementary Information (DOCX, 1398 KB)
