# Supplementary material for: Evaluation of a Validated Food Frequency Questionnaire for Self-Defined Vegans in the United States
Source: Nutrients. 2014 Jul 8;6(7):2523–39. doi: 10.3390/nu6072523 (PMC4113754; doi:10.3390/nu6072523)
Supplement: Supplementary File 1 — Supplementary Information (DOCX, 1398 KB) [file nutrients-06-02523-s001.docx]

**Supplementary Information**

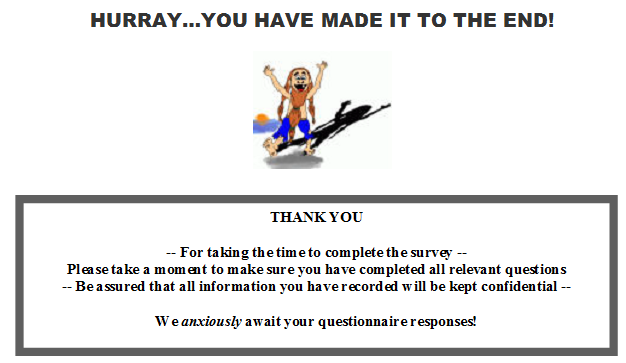

Appendix B

Scoring Instructions for the Vegan Food Frequency Questionnaire

1. The food frequency section of the questionnaire begins at question #41 and ends at
   question #291.
2. Scoring is based on the question, the 3 coded portion sizes, the 6 frequency options, and a weight factor.
3. The Score Formula is given below:

*FFQ nutrient intake score per day = (coded PF × coded FF × WF per question)/7*

Where ‘PF’ represents portion factor, ‘FF’ represents frequency factor, and ‘WF’ represents weight factor per food frequency question which is the unit amount of the nutrient for the middle portion size option.

1. Portion Factors:

(Portion code numbers 1, 2, & 3 are found at the bottom of each page in the FFQ); the middle portion size is the reference portion/weight. Different portion factor examples are given below.

| **FFQ Question** | **1^st^ Portion size** | **Code/(Factor)** | **Middle Portion size** | **Code/(Factor)** | **3^rd^ Portion size** | **Code/(Factor)** |
| --- | --- | --- | --- | --- | --- | --- |
| 41 | ½ cup | 1 = (0.67) | ¾ cup | 2 = (1) | 1 cup | 3 = (1.34) |
| 63 | 1/8 cup | 1 = (0.5) | ¼ cup | 2 = (1) | ½ cup | 3 = (2) |
| 73 | 2 tsp | 1 = (0.66) | 1 Tbsp | 2 = (1) | 2 Tbsp | 3 = (2) |
| 100 | 1 slice | 1 = (0.5) | 2 slices | 2 = (1) | 2 ½ slices | 3 = (1.5) |

1. Frequency Factors:

(Frequency code numbers are found at the bottom of each page in the FFQ)

| **Code** | **FFactor** | **Meaning** |
| --- | --- | --- |
| 4 | = 0 | Never or rarely |
| 5 | = 0.75 | 2 to 4 times per month |
| 6 | = 2.5 | 2 to 3 times per week |
| 7 | = 5 | 4 to 6 times per week |
| 8 | = 7 | once per day |
| 9 | = 14 | 2 to 3+ times per day |

1. Weight Factors:

This is the unit amount of the nutrient per the middle portion of each questionnaire food item.

E.g., the amount of zinc in ¾ cup kidney beans is 1.42 mg based on the diet analysis
program used.

1. Calculation Example:

| **FFQ nutrient intake score per day = (coded PF × coded FF × WF per question)/7** |
| --- |

If 1 cup (portion) of kidney beans is used 2 to 4 times per month (frequency); the zinc intake score from kidney beans per day = (1.34 × 0.75 × 1.42)/7 = **0.2 mg zinc intake per day**

© 2014 by the authors; licensee MDPI, Basel, Switzerland. This article is an open access article distributed under the terms and conditions of the Creative Commons Attribution license (http://creativecommons.org/licenses/by/3.0/).
